# Sex estimation using maxillary sinus volume for Chinese subjects based on cone-beam computed tomography

**DOI:** 10.1186/s12903-024-04010-5

**Published:** 2024-02-19

**Authors:** Zi-Xuan Wu, Wen-Qing Bu, Yu Tang, Yu-Xin Guo, Yu-Cheng Guo, Fei Wang, Hao-Tian Meng

**Affiliations:** 1 Key Laboratory of Shaanxi Province for Craniofacial Precision Medicine Research, College of Stomatology, Xi’an Jiaotong University, 98 XiWu Road, Xi’an, 710004 Shaanxi People’s Republic of China; 2 Department of Orthodontics, Stomatological Hospital of Xi’an Jiaotong University, 98 XiWu Road, Xi’an, 710004 Shaanxi People’s Republic of China; 3 College of Medicine and Forensics, Xi’an Jiaotong University Health Science Center, 76 West Yanta Road, Xi’an, 710004 Shaanxi People’s Republic of China

**Keywords:** Sex estimation, Forensic science, Maxillary sinus, Volume, Cone-beam computed tomography

## Abstract

**Background:**

Sex estimate is a key stage in forensic science for identifying individuals. Some anatomical structures may be useful for sex estimation since they retain their integrity even after highly severe events. However, few studies are focusing on the Chinese population. Some researchers used teeth for sex estimation, but comparison with maxillary sinus were lack. As a result, the objective of this research is to develop a sex estimation formula for the northwestern Chinese population by the volume of the maxillary sinus and compare with the accuracy of sex estimation based on teeth through cone-beam computed tomography (CBCT).

**Methods:**

CBCT images from 349 samples were used to establish and verify the formula. The volume of both the left and right maxillary sinuses was measured and examined for appropriate formula coefficients. To create the formula, we randomly picked 80% of the data as the training set and 20% of the samples as the testing set. Another set of samples, including 20 males and 20 females, were used to compare the accuracy of maxillary sinuses and teeth.

**Results:**

Overall, sex estimation accuracy by volume of the left maxillary sinus can reach 78.57%, while by the volume of the right maxillary sinus can reach 74.29%. The accuracy for females, which can reach 91.43% using the left maxillary sinus, was significantly higher than that for males, which was 65.71%. The result also shows that maxillary sinus volume was higher in males. The comparison with the available results using measurements of teeth for sex estimation performed by our group showed that the accuracy of sex estimation using canines volume was higher than the one using maxillary sinus volume, the accuracies based on mesiodistal diameter of canine and first molar were the same or lower than the volume of maxillary sinus.

**Conclusions:**

The study demonstrates that measurement of maxillary sinus volume based on CBCT scans was an available and alternative method for sex estimation. And we established a method to accurately assess the sex of the northwest Chinese population. The comparison with the results of teeth measurements made the conclusion more reliable.

**Supplementary Information:**

The online version contains supplementary material available at 10.1186/s12903-024-04010-5.

## Background

Individual identification plays an important role in forensic anthropological science. And sex estimation could be a necessary step in individual identification [1]. 

A lot of methods are available for estimating sex, such as bone measurement [2] and chromosome testing [3]. DNA testing has been widely used in sex estimation. However, there are disadvantages: requiring lots of time and manpower which doesn’t benefit the timeliness of cases, leading to failure of DNA testing if samples are in trace amounts, highly degraded or mixed, and requiring relevant large equipment which may be not available in some remote areas where have poor economic levels and lack of technological capability [4]. 

Thus, it is necessary to use the craniomaxillofacial hard tissue morphology of the remains to estimate sex. Usually, skeletal remains are used for sex estimation, since bones are relatively stable tissues of the human body [5]. There are plenty of craniomaxillofacial morphology measurements that showed satisfactory results in sex estimation such as tooth [6, 7], mandible [8, 9] and maxillary sinus. Among them, the maxillary sinuses may maintain their anatomical integrity in some extreme situations [5, 10, 11] Previous studies showed that the maxillary sinus exhibited anatomic variability between sex [10, 12–14]. Thus, measuring the anatomical structure of maxillary sinuses for sex estimation may have relatively high accuracy. Recently, there are increasing number of researchers interested in sex estimation related to maxillary sinus morphology due to their satisfactory results. The study of Wanzeler [15] showed a high accuracy of 84.66% for sex estimation using the volume of maxillary sinuses. Gomes et al. [16] also got a satisfactory result of 84% using three dimensions measurements of maxillary sinuses. Additionally, although plenty of researches independently used different features for sex estimation, there was lack of studies comparing the accuracies using different features.

Moreover, some researchers found that the size of the maxillary sinus develops with age increasing in patients who are less than 18 years [17, 18]. Samples who are below the age of 18 may obviously influence the result of sex estimation. So in this study, we selected patients over 18 years old for sex estimation.

Cone-beam computed tomography (CBCT) scan is one of the computed tomography (CT) imaging techniques that are focused on head and neck imaging [19], which generally costs less and has more compact in terms of equipment size [20]. There are some significant advantages of CBCT technology: good image quality and accuracy, scanning of three dimensions of hard tissues clearly, and low dose of radiation. Thus, CBCT scans are ideal for morphological research. What’s more, a large number of conditions in dental and medical fields suggest that CBCT examination can ensure the availability of images in cases that require the identification of individuals by comparing antemortem and postmortem records [21]. Nowadays, researchers gradually recognize the contribution of various imaging techniques, especially CBCT scans for forensic identification [22–25], and they performed some studies that ensure the validity and reliability of these techniques [26–29]. Therefore, CBCT scans are sure to acquire the accurate and clear three dimension of the maxillary sinus which we needed to focus on. And a tool to perform the measurements was required. 3D Slicer software can process and perform measurements on 3D medical images. There was plenty of research using 3D Slicer software as their volumetric measurement tool [30–32]. In this study, we also used 3D Slicer software to perform the volumetric measurements.

Moreover, there are studies shown that the sexual dimorphism of bone tissue structure is influenced by regional factors [33]. Several studies [12, 19, 34, 35] on sexual dimorphism of the human skeleton highlighted the need to establish anthropometric standards for different modern populations throughout the globe [16]. Since sex estimation is an important step to describe the biological profile and identify the individuals and there was a lack of research for the Chinese population, the purpose of this study is to establish a sex estimation method by measuring the volume of maxillary sinuses based on CBCT scans among northwestern Chinese adults population. Besides, because of the lacking of researches comparing the accuracies using different craniofacial structures for sex estimation, the comparison of the results of maxillary sinus and tooth will also be performed in this study.

## Methods

### Samples selection

In this study, we selected 349 samples in total (175 males and 174 females) aged between 18 and 56 years (Table 1) from the Department of Oral Radiology, Stomatology Hospital of Xi'an Jiaotong University. Besides, another set of samples with 15 males and 15 females who were aged above 18 were used as cross-validation set. All the CBCT scans were taken by the Cone-beam X-ray Computed Tomography System (KaVO 3D eXam I, USA), and exported into DICOM format. The current research was conducted after the approval of the Biomedical Ethics Committee of Xi’an Jiaotong University Health Science Center, China (No: [2021] 1473). The inclusion criteria of sample selection were as follow:

1. Northwestern Chinese population without trauma, surgery, deformities, systemic illnesses and malnutrition;
2. both left and right maxillary sinuses are captured completely;
3. the boundary between the maxillary sinus and surrounding tissues are visible;
4. no severe inflammation in the maxillary sinus;
5. no tooth breaking in the maxillary sinus.

**Table 1:** The age and sex distribution of the selected population

Age (y)	Male (n)	Female (n)	Total (n)
18–25	58	55	113
26–35	50	55	105
36–56	67	64	131
Total(n)	175	174	349

The following information was recorded: patient’s ID, name, chronological age, sex, date of birth and date of exposure.

Another set of samples, including 20 males and 20 females who were aged above 18, were collected for comparing the results of maxillary sinus using equations produced in this study with the results of teeth using equations produced from the study of Bu et al. [4].

### Volume measurement

**Fig. 1:**
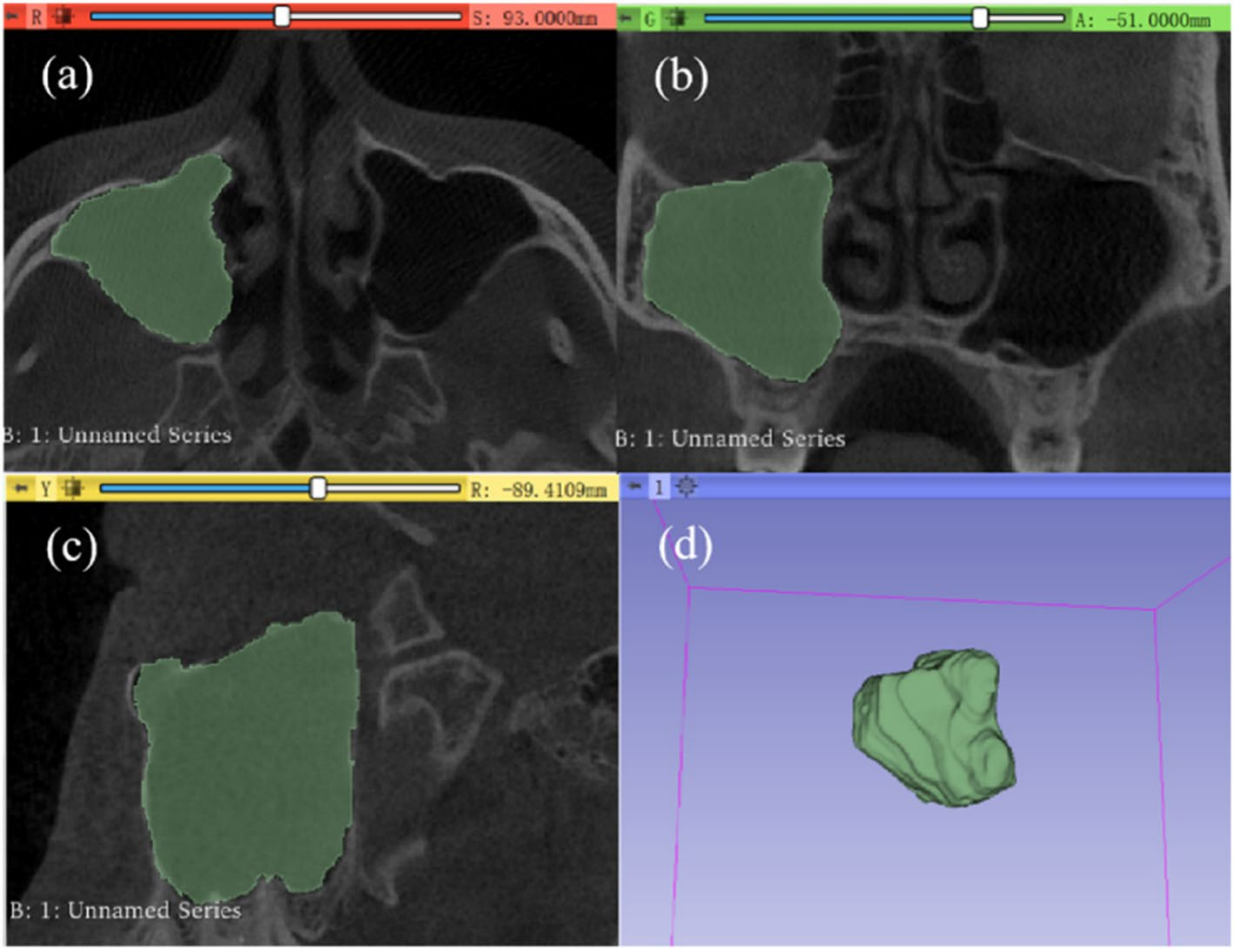
The measurement in the 3D Slicer software: **a** the sagittal direction of a slide completed tracing the boundary of the right maxillary sinus; **b** the vertical direction of a slide completed tracing the boundary of right maxillary sinus; **c** the lateral direction of a slide completed tracing the boundary of right maxillary sinus; **d** the 3D model of right maxillary sinus constructed after all the tracing work was finished

All the CBCT files were imported into 3D Slicer software (version 4.11.20210226, USA) [36] for measurement. The images were segmented into pieces for every 0.3 mm in the sagittal direction. The researcher traced the boundary of the maxillary sinus on each slide for every 5 pieces (1.5 mm). The cavity can be full-filled automatically after the tracing is finished. Then the software was used to synthesize the complete anatomical form of maxillary sinuses with the slides traced and calculate the volume (Fig. 1). The volumes were measured in both left and right maxillary sinuses.

### Reliable and repeatable test

Before the formal measurement, we randomly selected twenty samples in the preliminary experiment to test the inter- and intra-observer variation of measurements. Two researchers were required for the preliminary experiment. Both of them repeated the measurements at the one-month interval. After finishing the preliminary measurements, both the inter- and intra-class correlation coefficient test (ICC) results were analyzed and reached the acceptable level. Then they were allowed to start the standard measurements.

### Statistics analysis

All the statistical analyses were performed by IBM SPSS Statistics 24 software (IBM® SPSS® Statistics, Armonk, NY). We randomly grouped all the data into training set and testing set with a ratio of 8:2 (Table 2). The volume measurements and normality test were conducted for all the data to obtain the descriptive indicators of the measurement results including mean value, minimum value, maximum value and standard deviation (SD). The Spearman’s correlation test was used to check whether there were linear correlations between age and maxillary sinus volume. The Pearson’s correlation test was used to check the correlation strength between age and maxillary sinus volume. The independent t-test was used to check whether there were differences between sex and maxillary sinus volume. The paired t-test was used to check whether there were volumetric differences between the left and right maxillary sinus. The training set was used to perform the logistic regression analysis and obtain the equation. All the equations should pass the Hosmer and Lemeshow test. Then the testing set was used to validate the obtained equation and get the accuracy (ACC). Besides, the sensitivity, specificity, positive predictive value (PPV) and negative predictive value (NPV) of the equation were also calculated (Table 3).

**Table 2:** The distribution of the sample studied

	Training Set (n)	Testing Set (n)	Total (n)
Male	140	35	175
Female	139	35	174
Total	279	70	349

**Table 3:** Definition and equations of the values

Value	Definition	Equation
ACC	The proportion of samples correctly predicted	$ACC=\frac{Samples\;tested\;as\;male\;that\;is\;male\;+\;Samples\;tested\;as\;female\;that\;is\;female}{Total\;Samples\;Size}\times100\%$
Sensitivity	The proportion of samples tested as male	$Sensitivity=\frac{Samples\;tested\;as\;male\;that\;is\;male\;}{Male\;Sample\;Size}\times100\%$
Specificity	The proportion of samples tested as female	$Specificity=\frac{Samples\;tested\;as\;female\;that\;is\;female}{Female\;Sample\;Size}\times100\%$
PPV	The proportion of samples judged to be male by the test that is male	$PPV=\frac{Samples\;tested\;as\;male\;that\;is\;male}{Samples\;tested\;as\;male}\times100\%$
NPV	The proportion of samples judged to be female by the test that is female	$NPV=\frac{Samples\;tested\;as\;female\;that\;is\;female}{Samples\;tested\;as\;female}\times100\%$

## Results

### Reliable and repeatable test

In the preliminary experiment, the reliability analysis showed that the ICC test revealed excellent results for both inter-observer and intra-observer measurements (ICC = 0.998 and ICC = 0.997, *p* < 0.05).

### Descriptive statistics

**Fig. 2:**
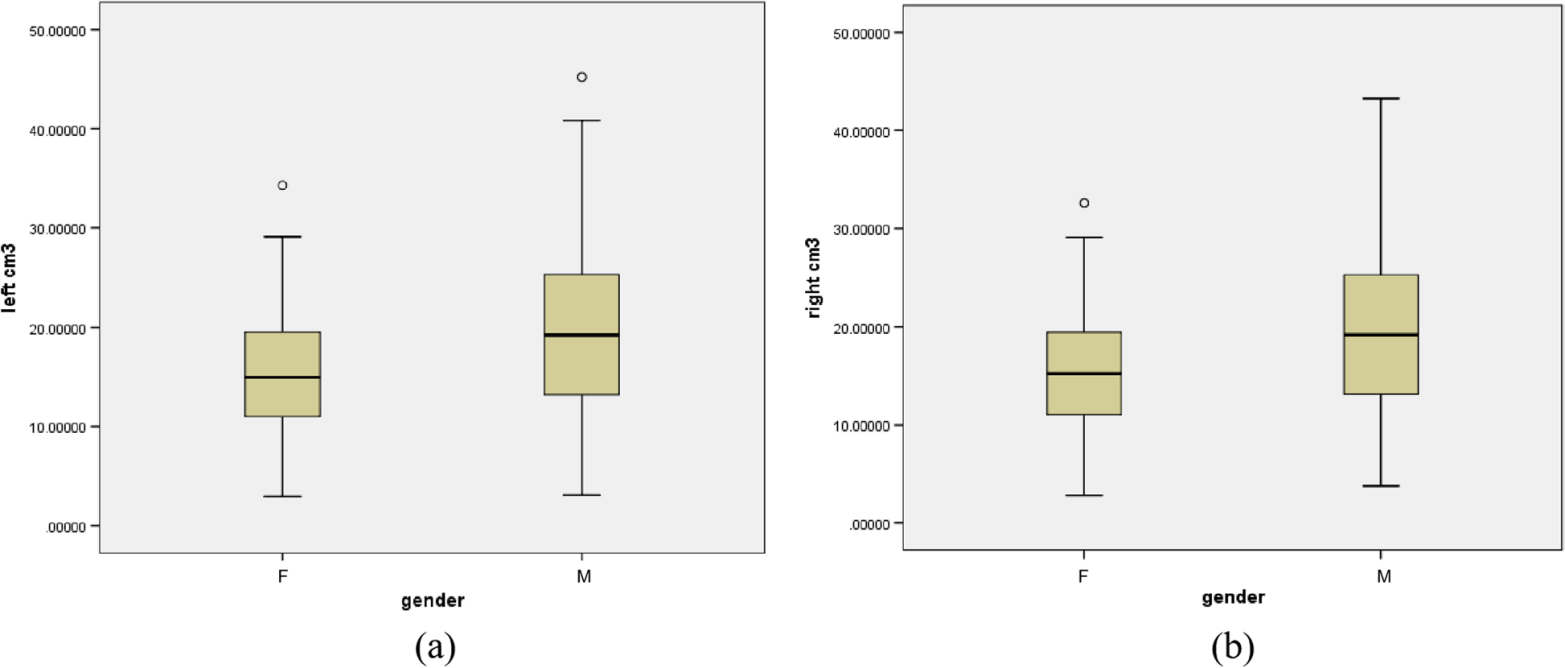
The stem-and-leaf plots of the research: **a** The stem-and-leaf plot of left maxillary sinuses volume; **b** The stem-and-leaf plot of right maxillary sinuses volume

The descriptive analysis showed that all the volumes were significantly higher in males (Table 4; Fig. 2). And the result of the Kolmogorov-Smirnov test indicated that all the sample sets conformed to a normal distribution (*p* > 0.05) (Table 5).

**Table 4:** The descriptive statistics of the research

Sex	Index	Mean	Minimum	Maximum	SD
Male	VL (cm^3^)	19.7083	3.0821	45.2180	8.2831
	VR (cm^3^)	19.8221	3.7886	43.2525	8.3478
Female	VL (cm^3^)	15.5571	2.9644	34.2827	5.6204
	VR (cm^3^)	15.4243	2.7950	32.5904	5.7688

*VL* Volume of the left maxillary sinus, *VR *Volume of the right maxillary sinus

**Table 5:** Kolmogorov-Smirnov test, t-test, Pearson correlation test results

Independent Variable	Kolmogorov-Smirnov test		Independent t-test with sex		Pearson correlation test with age	
	Kolmogorov-Smirnov Z	*p* value	t	*p* value	Pearson correlation coefficient	*p* value
VL (cm^3^)	0.047	0.200	-5.475	0.000	-0.424	0.000
VR (cm^3^)	0.049	0.200	-5.722	0.000	-0.385	0.000

### Correlations

The results of Spearman’s correlation test showed that there were linear correlations between age and maxillary sinus volume (*p* < 0.05). Pearson’s correlation test suggested that the correlations between age and the volume of the left and right maxillary sinus were weak to moderate (r_VL_=-0.424, *p* < 0.01 and r_VR_=-0.385, *p* < 0.01) (Table 5). According to the independent t-test, there were significant differences between both two sides of maxillary sinuses volume from different sex (*p* < 0.01) (Table 5). And according to the paired t-test, there was no significant difference between left and right maxillary sinuses volume (*p* = 0.952).

### Logistic regression analysis

Two variables, including the volume of the left maxillary sinus (VL) and the volume of the right maxillary sinus (VR), were used to set up sex estimating equations by using logistic regression analysis. The analysis suggested that all the equations had passed Hosmer and Lemeshow test (*p* > 0.05). But when using both VL and VR as variables in one equation, the coefficients were not available (*p*_*VL*_=0.409 and *p*_*VR*_=0.504). The coefficients of variables in the equations established with VL and VR independently were available (*p* < 0.01).

We tried to add age as a variable into the models. But we found that after adding age as a variable, the Akaike information criterion (AIC) and Bayesian information criterion (BIC) values of both VL and VR models did not significantly decrease (VL without age: AIC = 375.956, BIC = 383.218; VL with age: AIC = 374.590, BIC = 385.483; VR without age: AIC = 376.189, BIC = 383.451; VR with age: AIC = 375.481, BIC = 386.375.). Thus, age could be excluded from equations set up for sex estimation. Besides, considering the potential random effects of the individuals, we tried to create the mixed models with random intercepts and slopes. However, the results of AIC and BIC values of these models were extremely larger than the models with fixed intercepts and slopes (VL with random intercept and slope: AIC = 1204.826, BIC = 1212.030; VR with random intercept and slope: AIC = 1204.344, BIC = 1211.549). Therefore, we chose the models with fixed intercepts and slopes.

Then the established equations (Table 6) were substituted into the testing set for ACC. 78.57% of samples in total were correctly assigned to their sex group using the volume of left maxillary sinuses, and 74.29% of samples in total were correctly estimated with the volume of right maxillary sinuses. What’s more, the results also showed that the specificity and PPV value were higher than 85% both using the volume of the left and right maxillary sinuses, but sensitivity and NPV value were lower than 75%. The cross-validation set suggested similar results (Table 6).

**Table 6:** Sex estimation equations based on different values

	Independent Variable	Equation	SD	Accuracy	Sensitivity		Specificity	PPV	NPV
Original	VL cm^3^	Logit(p)=-1.118 + 0.067X	0.2311	78.57%	65.71%		91.43%	88.46%	72.73%
	VR cm^3^	Logit(p)=-1.099 + 0.065X	0.2892	74.29%	51.43%		97.14%	94.74%	65.38%
Cross-Validation	VL cm^3^	Logit(p)=-1.118 + 0.067X	0.2189	76.67%	66.67%		86.67%	83.33%	72.22%
	VR cm^3^	Logit(p)=-1.099 + 0.065X	0.2190	73.33%	53.33%		93.33%	88.89%	66.67%

*Logit(p)* the probability value obtained by the equation, *VL *the volume of the left maxillary sinus, *VR *the volume of the right maxillary sinus; sensitivity, the proportion of samples tested as male; specificity, the proportion of samples tested as female, *PPV *the proportion of samples judged to be male by the test that is actually male, *NPV *the proportion of samples judged to be female by the test that is actually female

### Comparison with measurement of teeth

The results (Table 7) showed that the ACC using indexes including the volume of the left maxillary canine (V23), the volume of the left mandibular canine (V33) and V23 + V33 were higher than the ones using the volume of maxillary sinus. The ACC of the mesiodistal diameter of the left maxillary canine (L23) was the same as VR but higher than VL. The ACC of the mesiodistal diameter of the left mandibular first molar (L36) was lower than VR but higher than VL. The ACC of L23 + L36 was lower than the ones using the volume of maxillary sinus.

**Table 7:** Sex Estimation equations based on maxillary sinus and tooth indexes

Independent Variable		Equation	SD	Accuracy	Sensitivity	Specificity	PPV	NPV
Maxillary Sinus	VL cm^3^	Logit(p)=-1.118 + 0.067VL	0.1071	67.50%	60.00%	75.00%	70.59%	65.22%
	VR cm^3^	Logit(p)=-1.099 + 0.065VR	0.1159	72.50%	65.00%	80.00%	76.47%	69.57%
Tooth [4]	V23 cm^3^	Logit(p)=-8.497 + 14.118V23	0.7601	77.50%	90.00%	65.00%	72.00%	86.67%
	V33 cm^3^	Logit(p)=-6.003 + 11.367V33	0.5176	77.50%	80.00%	75.00%	76.19%	78.95%
	V23 + V33 cm^3^	Logit(p)=-8.914 + 10.457V23 + 4.923V33	0.8535	77.50%	90.00%	65.00%	72.00%	86.67%
	L23 mm	Logit(p)=-12.471 + 1.658L23	0.0014	72.50%	75.00%	70.00%	71.43%	73.68%
	L36 mm	Logit(p)=-19.68 + 1.798L36	0.0318	70.00%	70.00%	70.00%	70.00%	70.00%
	L23 + L36 mm	Logit(p)=-29.38 + 1.501L23 + 1.657L36	0.4882	65.00%	70.00%	60.00%	63.64%	66.67%

*Logit(p)* the probability value obtained by the equation, *VL *the volume of the left maxillary sinus, *VR *the volume of the right maxillary sinus; sensitivity, the proportion of samples tested as male; specificity, the proportion of samples tested as female, *PPV *the proportion of samples judged to be male by the test that is actually male, *NPV *the proportion of samples judged to be female by the test that is actually female

## Discussion

Through this research, we were going to establish a sex estimation method by measuring the volume of maxillary sinuses based on CBCT scans among northwestern Chinese population. Eventually, we produced the results that the equations developed in this research showed an accuracy of 78.57% for sex estimation in the northwest Chinese population who were no less than 18 years. Therefore, based on the results, we had the following discussion.

The independent t-test between maxillary sinus volume and sex showed the result that there are significant differences between different sex in both two sides of the maxillary sinus volume, which confirmed that the volume of the maxillary sinus has sexual dimorphism.

The result of the Pearson correlation test showed that the correlation between age and maxillary sinus volume is weak. And the comparison results of the AIC and BIC values between whether adding age into the model as a variable suggested that the variable, age, could be excluded from the equations. The height of the maxillary sinus has a constant, steady increase from birth to at least the age of 18. The increase of the width and depth of the maxillary sinus decreases with time to the point where there is no significant growth after 12 years of age [17]. Thus, the results were stable based on the samples above 18 years old in this study. Besides, the result of the independent t-test between left and right maxillary sinus volume suggested that there is no significant volumetric difference between left and right maxillary sinus, which explained that the equations established in this study could be used in both left and right maxillary sinuses.

Although the results showed a satisfactory accuracy of 78.57% for VL and 74.29% for VR, the other formula performance values (sensitivity, specificity, PPV and NPV) suggested that the disparity between males and females was enormous, with prediction accuracy above 95% in females, but lower than 55% in males. The low sensitivity in this study suggested that there were only about 60% of males who were correctly predicted using the volume of the maxillary sinus. But the high specificity showed that most females can be correctly predicted. Besides, the high PPV value suggested that most of samples were males in reality while the results of prediction were males in this study. On the contrary, the low NPV value showed that if the results prediction of patients were females, there were still plenty of them were males. Both the study of Wanzeler [15] and Gomes [16] had similar results. The result of this study showed that the maxillary sinuses were significantly larger in males than in females. And the maxillary sinus volume of males was more dispersed than that of females. Although males had significant larger maxillary sinuses than females, there were still a number of males might be predicted as females for their maxillary sinus were relatively small. The study of Radulesco [37] and Sharma [38] also produced similar results. The most relative studies also demonstrated this point, which also suggested that the differences were especially significant in 18–24 [15, 16, 37, 39]. And the maxillary sinuses of females who were above the age of 24 were also smaller than the ones of males, but the differences were not significant [40]. Besides, several studies showed that the volume of maxillary sinus decreases as age increases [41, 42], especially in the completely and partially edentulous patients [41]. In this study, although the influence of age has already excluded according to the statistical analysis, the aging changes still might impact the accuracy of sex estimation based on previous researches.

There are a lot of studies showed good performance in sex estimation by maxillary sinus volumetric measurements. And the sexual dimorphism of maxillary sinus morphology could be influenced by regional disparity [33]. Ekizoglu et al. [13] found that there were 77.15% of samples which could be correctly predicted using the volume of maxillary sinus in Turkey. Wanzeler et al. [15] showed a high accuracy of 84.66% using volumetric measurements of maxillary sinus in Brazilian population. In addition, the study by Prabhat [43] had an accuracy of 83.3% based on the equation which included volumetric measurements of maxillary sinus in India. Radulesco et al. [37] got an accuracy of 68% using maxillary sinus volume in France. And there are also some studies using some other measurements of maxillary sinus. The study of Gomes [16] showed an accuracy above 84% using an equation set up with several measurements of maxillary sinus in Brazil. Dangore-Khasbage et al. [44] have successfully predicted 86% of patients using the anterolateral angle of the maxillary sinus in Indian population. Mathew et al. [45] also produced a satisfactory result, which was 80% on average, using three dimensions, vertical diameter, sagittal diameter and transverse diameter, of the maxillary sinus from Indian samples. The research of Gamba [19] suggested an accuracy of 75% using height and length of maxillary sinuses for sex estimation in the Dutch population. However, there is a lack of research from the Chinese population. Therefore, it is practical and important to estimate sex using maxillary sinus based on Chinese population. Eventually, the results of the present study were basically same to studies mentioned above.

Comparing with the accuracy for sex estimation between maxillary sinus and tooth equations, we found that the accuracy of the maxillary sinus volume was as satisfied as the measurement of tooth on the whole. However, age increasing and some oral diseases may lead to tooth loss or prosthodontic therapy, which may influence the capability of sex estimation with tooth. Besides, using tooth for sex estimation is not completely accurate. It is possible to associate maxillary sinus with tooth for sex estimation, which may further improve the reliability of sex estimation.

In addition to maxillary sinus, some researchers also use some other craniomaxillofacial hard tissue morphology for sex estimation. Previous studies suggested that dimensions of canines [6], frontal sinuses [15], sphenoid sinuses [15] and mandibular structures [9] had convinced accuracies, which were 76.2%, 94.48%, 77.91% and 76%-86% respectively. Also, some other hard tissues which are not craniomaxillofacial structures also attract researchers who are interested in sex estimation. Researchers using hyoid bones [46] and the second cervical vertebra [47] for sex estimation separately predicted 78.8% and 86.7%-89.7% of cases correctly. As is shown above, the accuracy using the volume of maxillary sinus for sex estimation in this study were basically same as those using other structures, the volume of maxillary sinus has already demonstrated as a valid method for sex estimation.

There are still plenty of limitations in this study. Firstly, although 3D Slicer has been confirmed as a valid tool to perform measurement tasks, it is time-consuming to use manual method to finish all the measurements and the errors produced from measurements cannot be avoided after all, which obviously decreased work efficiency. Secondly, although the age influence was excluded based on Pearson’s correlation test, the aging change of maxillary sinus still may affect the accuracy of the volume as a sex estimation index. So, in future studies, some automatic and artificial intelligent methods may be considered to make the measurements easier and further increase the accuracy of sex estimation. In addition, only the northwest Chinese population were analyzed in this study, while people from other region also required sex estimation. And the sample size of this research could be expanded. Thus, larger sample sizes and wider regional coverage are needed for further researches.

## Conclusions

In this study, the equations developed through measuring the volume of the maxillary sinus using CBCT scans showed an accuracy of 78.57% for sex estimation in the northwest Chinese adult population who were no less than 18 years, which would provide reference in the field of individual identification. The comparison of accuracy for sex estimation between measurements in the maxillary sinuses and teeth made the conclusion more reliable.

### Supplementary Information

**Supplementary material 1.**

## Data Availability

The datasets used and analyzed during the current study are available from the corresponding author on reasonable request.

## References

[1] Nagare SP, Chaudhari RS, Birangane RS, Parkarwar PC (2018). Sex determination in forensic identification, a review. J Forensic Dent Sci.

[2] Kruger GC, L’Abbe EN, Stull KE (2017). Sex estimation from the long bones of modern South africans. Int J Legal Med.

[3] Babu R, Shah U (2021). Gender identity disorder (GID) in adolescents and adults with differences of sex development (DSD): a systematic review and meta-analysis. J Pediatr Urol.

[4] Bu W, Ji L, Han M, Wu Z, Sultan B, Chen T, Tang Y, Guo Y, Wang F. Accuracy comparison of tooth volume and mesiodistal diameter measurements for sex dimorphism based on cone-beam computed tomography: a study for the northern Chinese population. Forensic Sci Res. 2023;8(2):133–9. 10.1093/fsr/owad004 PMC10445665 37621453

[5] Uthman AT, Al-Rawi NH, Al-Naaimi AS, Al-Timimi JF (2011). Evaluation of maxillary sinus dimensions in gender determination using helical CT scanning. J Forensic Sci.

[6] Daniele G, Matilde SA, Maria M, Rafael RV, Milagros AM (2020). Sex estimation by tooth dimension in a contemporary Spanish population. Forensic Sci Int.

[7] Kanchan T, Chugh V, Chugh A, Setia P, Shedge R, Krishan K (2021). Estimation of sex from Dental Arch dimensions: an odontometric analysis. J Craniofac Surg.

[8] Dietrichkeit Pereira JG, Lima KF, Alves da Silva RH (2020). Mandibular measurements for sex and age estimation in Brazilian sampling. Acta Stomatol Croat.

[9] Lopez-Capp TT, Rynn C, Wilkinson C, de Paiva LAS, Michel-Crosato E, Biazevic MGH (2018). Discriminant analysis of mandibular measurements for the estimation of sex in a modern Brazilian sample. Int J Legal Med.

[10] Amin MF, Hassan EI (2012). Sex identification in Egyptian population using Multidetector Computed Tomography of the maxillary sinus. J Forensic Leg Med.

[11] Akhlaghi M, Bakhtavar K, Kamali A, Maarefdoost J, Sheikhazadi A, Mousavi F, Saberi Anary SH, Sheikhazadi E (2017). The diagnostic value of anthropometric indices of maxillary sinuses for sex determination using CT-scan images in Iranian adults: a cross-sectional study. J Forensic Leg Med.

[12] Paknahad M, Shahidi S, Zarei Z (2017). Sexual dimorphism of Maxillary Sinus dimensions using Cone-Beam Computed Tomography. J Forensic Sci.

[13] Ekizoglu O, Inci E, Hocaoglu E, Sayin I, Kayhan FT, Can IO (2014). The use of maxillary sinus dimensions in gender determination: a thin-slice multidetector computed tomography assisted morphometric study. J Craniofac Surg.

[14] Teke HY, Duran S, Canturk N, Canturk G (2007). Determination of gender by measuring the size of the maxillary sinuses in computerized tomography scans. Surg Radiol Anat.

[15] Wanzeler AMV, Alves-Junior SM, Ayres L, da Costa Prestes MC, Gomes JT, Tuji FM (2019). Sex estimation using paranasal sinus discriminant analysis: a new approach via cone beam computerized tomography volume analysis. Int J Legal Med.

[16] Gomes AF, Gamba TD, Yamasaki MC, Groppo FC, Neto FH, Possobon RD (2019). Development and validation of a formula based on maxillary sinus measurements as a tool for sex estimation: a cone beam computed tomography study. Int J Legal Med.

[17] Bhushan B, Rychlik K, Schroeder JW (2016). Development of the maxillary sinus in infants and children. Int J Pediatr Otorhinolaryngol.

[18] Degermenci M, Ertekin T, Ulger H, Acer N, Coskun A (2016). The Age-Related Development of Maxillary Sinus in Children. J Craniofac Surg.

[19] Gamba TO, Yamasaki MC, Groppo FC, da Silveira HLD, Boscolo SMA, Sanderink GCH, Berkhout WER (2017). Validation study of a new method for sexual prediction based on CBCT analysis of maxillary sinus and mandibular canal. Arch Oral Biol.

[20] Sarment DP, Christensen AM (2014). The use of cone beam computed tomography in forensic radiology. J Forensic Radiol Imaging.

[21] Tambawala SS, Karjodkar FR, Sansare K, Prakash N (2016). Sexual dimorphism of maxillary sinus using cone beam computed tomography. Egypt J Forensic Sci.

[22] Leth PM (2009). Computerized tomography used as a routine procedure at postmortem investigations. Am J Forensic Med Pathol.

[23] Thali MJ, Braun M, Wirth J, Vock P, Dirnhofer R (2003). 3D surface and body documentation in forensic medicine: 3-D/CAD photogrammetry merged with 3D radiological scanning. J Forensic Sci.

[24] Thomsen AH, Jurik AG, Uhrenholt L, Vesterby A (2009). An alternative approach to computerized tomography (CT) in forensic pathology. Forensic Sci Int.

[25] Thali MJ, Yen K, Schweitzer W, Vock P, Boesch C, Ozdoba C, Schroth G, Ith M, Sonnenschein M, Doernhoefer T (2003). Virtopsy, a new imaging horizon in forensic pathology: virtual autopsy by postmortem multislice computed tomography (MSCT) and magnetic resonance imaging (MRI)--a feasibility study. J Forensic Sci.

[26] Uysal S, Gokharman D, Kacar M, Tuncbilek I, Kosa U (2005). Estimation of sex by 3D CT measurements of the foramen magnum. J Forensic Sci.

[27] Thali MJ, Yen K, Plattner T, Schweitzer W, Vock P, Ozdoba C, Dirnhofer R (2002). Charred body: virtual autopsy with multi-slice computed tomography and magnetic resonance imaging. J Forensic Sci.

[28] Verhoff MA, Ramsthaler F, Krahahn J, Deml U, Gille RJ, Grabherr S, Thali MJ, Kreutz K (2008). Digital forensic osteology–possibilities in cooperation with the Virtopsy project. Forensic Sci Int.

[29] Ramsthaler F, Kettner M, Gehl A, Verhoff MA (2010). Digital forensic osteology: morphological sexing of skeletal remains using volume-rendered cranial CT scans. Forensic Sci Int.

[30] Velazquez ER, Parmar C, Jermoumi M, Mak RH, van Baardwijk A, Fennessy FM, Lewis JH, De Ruysscher D, Kikinis R, Lambin P (2013). Volumetric CT-based segmentation of NSCLC using 3D-Slicer. Sci Rep.

[31] Egger J, Kapur T, Nimsky C, Kikinis R (2012). Pituitary adenoma volumetry with 3D slicer. PLoS ONE.

[32] Balbi M, Conti C, Imeri G, Caroli A, Surace A, Corsi A, Mercanzin E, Arrigoni A, Villa G, Di Marco F (2021). Post-discharge chest CT findings and pulmonary function tests in severe COVID-19 patients. Eur J Radiol.

[33] Petaros A, Garvin HM, Sholts SB, Schlager S, Warmlander S (2017). Sexual dimorphism and regional variation in human frontal bone inclination measured via digital 3D models. Leg Med (Tokyo).

[34] Gulhan O, Harrison K, Kiris A (2015). A new computer-tomography-based method of sex estimation: development of Turkish population-specific standards. Forensic Sci Int.

[35] Shehri FA, Soliman KE (2015). Determination of sex from radiographic measurements of the humerus by discriminant function analysis in Saudi population, Qassim region, KSA. Forensic Sci Int.

[36] Fedorov A, Beichel R, Kalpathy-Cramer J, Finet J, Fillion-Robin JC, Pujol S, Bauer C, Jennings D, Fennessy F, Sonka M (2012). 3D slicer as an image computing platform for the quantitative Imaging Network. Magn Reson Imaging.

[37] Radulesco T, Michel J, Mancini J, Dessi P, Adalian P (2018). Sex estimation from human cranium: forensic and anthropological interest of Maxillary Sinus volumes. J Forensic Sci.

[38] Sharma SK, Jehan M, Kumar A (2014). Measurements of maxillary sinus volume and dimensions by computed tomography scan for gender determination. J Anat Soc India.

[39] Nunes Rocha MF, Dietrichkeit Pereira JG, Alves da Silva RH (2021). Sex estimation by maxillary sinus using computed tomography: a systematic review. J Forensic Odontostomatol.

[40] Aktuna Belgin C, Colak M, Adiguzel O, Akkus Z, Orhan K (2019). Three-dimensional evaluation of maxillary sinus volume in different age and sex groups using CBCT. Eur Arch Otorhinolaryngol.

[41] Velasco-Torres M, Padial-Molina M, Avila-Ortiz G, Garcia-Delgado R, O’Valle F, Catena A, Galindo-Moreno P (2017). Maxillary sinus dimensions decrease as age and tooth loss increase. Implant Dent.

[42] Jun BC, Song SW, Park CS, Lee DH, Cho KJ, Cho JH (2005). The analysis of maxillary sinus aeration according to aging process; volume assessment by 3-dimensional reconstruction by high-resolutional CT scanning. Otolaryngol Head Neck Surg.

[43] Prabhat M, Rai S, Kaur M, Prabhat K, Bhatnagar P, Panjwani S (2016). Computed tomography based forensic gender determination by measuring the size and volume of the maxillary sinuses. J Forensic Dent Sci.

[44] Dangore-Khasbage S, Bhowate R. Utility of the morphometry of the maxillary sinuses for gender determination by using computed tomography. Dent Med Probl. 2018;55(4):411–7. 10.17219/dmp/99622 30648366

[45] Anju M, Jacob LE. 3D evaluation of maxillary sinus in gender determination: A cone beam computed tomography study. J Indian Acad Oral Med Radiol. 2020;32(4):384–9.

[46] Balseven-Odabasi A, Yalcinozan E, Keten A, Akcan R, Tumer AR, Onan A, Canturk N, Odabasi O, Hakan Dinc A (2013). Age and sex estimation by metric measurements and fusion of hyoid bone in a Turkish population. J Forensic Leg Med.

[47] Gama I, Navega D, Cunha E (2015). Sex estimation using the second cervical vertebra: a morphometric analysis in a documented Portuguese skeletal sample. Int J Legal Med.

