# Supplementary material for: Sex estimation using maxillary sinus volume for Chinese subjects based on cone-beam computed tomography
Source: BMC Oral Health. 2024 Feb 19;24:253. doi: 10.1186/s12903-024-04010-5 (PMC10875788; doi:10.1186/s12903-024-04010-5)
Supplement: Supplementary file 1 — Supplementary material 1. [file 12903_2024_4010_MOESM1_ESM.docx]

(a)**
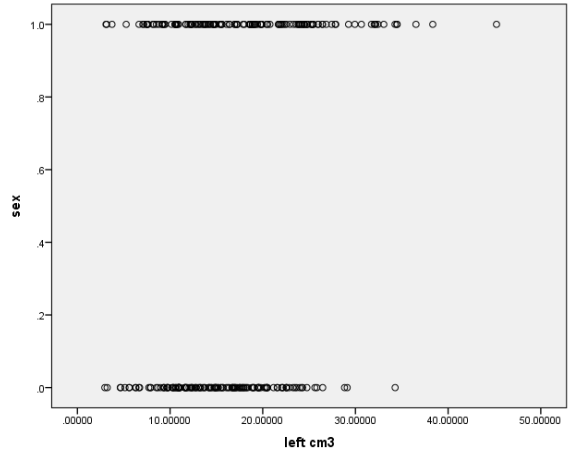
** (b)**
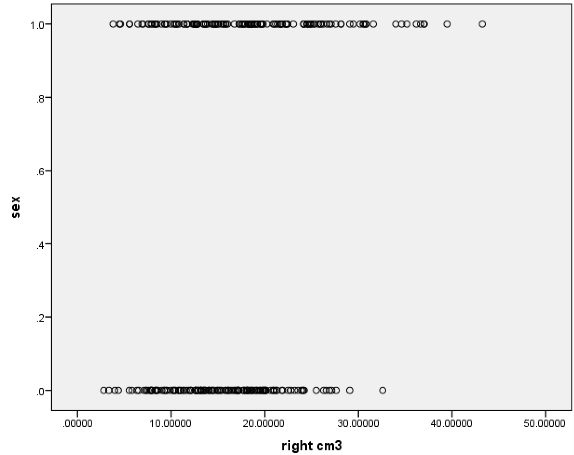
**

**Supplementary Fig. 1** The scatter plots of this research. The X-axis presented the volume of maxillary sinus. The Y-axis presented the sex. The males were defined as “1” and females were defined as “0”. (a) The scatter plot of the correlations between left maxillary sinus volume and sex. (b) The scatter plot of the correlations between right maxillary sinus volume and sex.
